# Spatial Distribution and Ecological Risks of the Potentially-Toxic Elements in the Surface Sediments of Lake Bosten, China

**DOI:** 10.3390/toxics8030077

**Published:** 2020-09-22

**Authors:** Long Ma, Jilili Abuduwaili, Wen Liu

**Affiliations:** 1 State Key Laboratory of Desert and Oasis Ecology, Xinjiang Institute of Ecology and Geography, Chinese Academy of Sciences, Urumqi 830011, China; jilil@ms.xjb.ac.cn (J.A.); liuwen@ms.xjb.ac.cn (W.L.); 2 Research Center for Ecology and Environment of Central Asia, Chinese Academy of Sciences, Urumqi 830011, China; 3 University of Chinese Academy of Sciences, Beijing 100049, China

**Keywords:** spatial distribution, ecological risk, potentially-toxic elements, inland water, lake sediments, China

## Abstract

Aiming at the pollution and ecological hazards of the lake sediments of Bosten Lake, once China’s largest inland lake, the spatial distribution and influencing factors of the potentially-toxic elements in its surface sediments were studied with the methods of spatial autocorrelation, two-way cluster analysis, and redundancy analysis. Finally, based on the background value of potentially-toxic elements extracted from a sediment core, a comprehensive evaluation of the risk of these potentially-toxic elements was conducted with the potential-ecological-risk index and the pollution-load index. With data on the grain size, bulk-rock composition, and organic matter content, this comprehensive analysis suggested that with the enrichment of authigenic carbonate minerals, the content of potentially-toxic elements exhibited distinctive characteristics representative of arid regions with lower values than those in humid region. All potentially-toxic elements revealed a significant spatial autocorrelation, and high-value areas mainly occurred in the middle and southwest. The content of potentially-toxic elements is related to Al_2_O3, K_2_O, Fe_2_O_3_, TiO_2_, MgO, and MnO, and the storage medium of potentially-toxic elements mainly consists of small particles with a grain size <16 μm. The pollution load index (PLI) for the whole lake due to the potentially-toxic elements was 1.31, and the surface area with a PLI higher than 1 and a moderate pollution level accounted for 87.2% of the total lake area. The research conclusions have an important scientific value for future lake ecological quality assessment and lake environment governance.

## 1. Introduction

Lake sediments are subject to material accumulation in watersheds and reflect the source–sink process [1]; lake sediments are also an important part of the lake environment and have special importance for ecological processes [2,3]. Lake sediments possess a high holding capacity for various pollutants. Most of the pollutants that enter water through various channels are quickly transferred to sediments, and the pollutants in sediments are again released under the conditions of environmental change and, therefore, become a secondary pollution source of the overlying water [4,5]. Among the numerous pollutants [6,7], potentially-toxic elements are some of the most serious pollutants due to their toxicity, durability, non-degradability, and bioaccumulation properties [8]. Potentially-toxic elements (PTEs) originate from the input of natural and human activities, and the main pathway of entering a water body is direct input through surface runoff, atmospheric deposition, and human activities [9,10]. Against the natural background, rivers transport the weathered products of rock minerals that are the main source of the PTEs in sediments. With the advancement of society, the process of urban development, agricultural and industrial activities in lake basins, such as urban transportation, fossil fuel combustion, mining, and metal smelting, and the use of fertilizers and pesticides have largely resulted in aquatic environmental pollution. PTEs impose a toxic or chronic effect on the organisms in lakes, especially benthic organisms [11], and accumulate in organisms and cause harm to humans [12]. Therefore, the geochemical characteristics, source analysis, and pollution assessment for PTEs in lake sediments have an important practical significance.

Lake Bosten was once the largest inland freshwater lake in China, located in Bohu County, the Xinjiang Uygur Autonomous Region [13]. It not only plays an important role in the ecological regulation of the lake basin but also directly affects the sustainable development of the economic and social environments of the region [14]. Large-scale industrial and agricultural development activities and the rapid increase in the population of the Yanqi Basin, Bosten Lake, have caused pollution via high-salinity farmland drainage and industrial sewage discharge, and regional pollution is gradually increasing [15,16]. Researchers have previously conducted fruitful research on the modern environment of Lake Bosten [17,18] and environmental changes over the historical period have been reconstructed [19,20]. Current research on the lake surface sediments of Bosten Lake has mainly focused on organic pollution [21,22], organic carbon [23], bacterial communities [24], chironomids [25], and PTEs [26,27]. Previous research on potentially-toxic elements has mainly centered on their statistical characteristics, but there are no studies on their spatial distribution characteristics [26]. In addition to the spatial distribution of PTEs, research on the relationship among the contents of potentially-toxic elements and environmental factors (such as the grain size and total organic matter) in the surface sediments of Bosten Lake is not available.

This research aimed to reveal whether human activities have significantly enhanced the enrichment of PTEs in lake sediments of underdeveloped areas in China, and to provide an approach to discussing the factors that influence PTEs through a comprehensive analysis of the grain size, bulk-rock composition, and organic matter content in lake sediments. Twenty-two points in the surface layer (0–5 cm) of Bosten Lake were sampled to analyze the content of PTEs, grain size, total organic carbon (TOC), and bulk-rock composition. The spatial distribution and influencing factors of the PTEs and their potential ecological risks were revealed with the methods of spatial autocorrelation, cluster analysis, and redundancy analysis, which will be of scientific value for future lake ecological quality assessment and lake environment governance.

## 2. Materials and Methods

### 2.1. Sampling and Analysis

**Figure 1:**
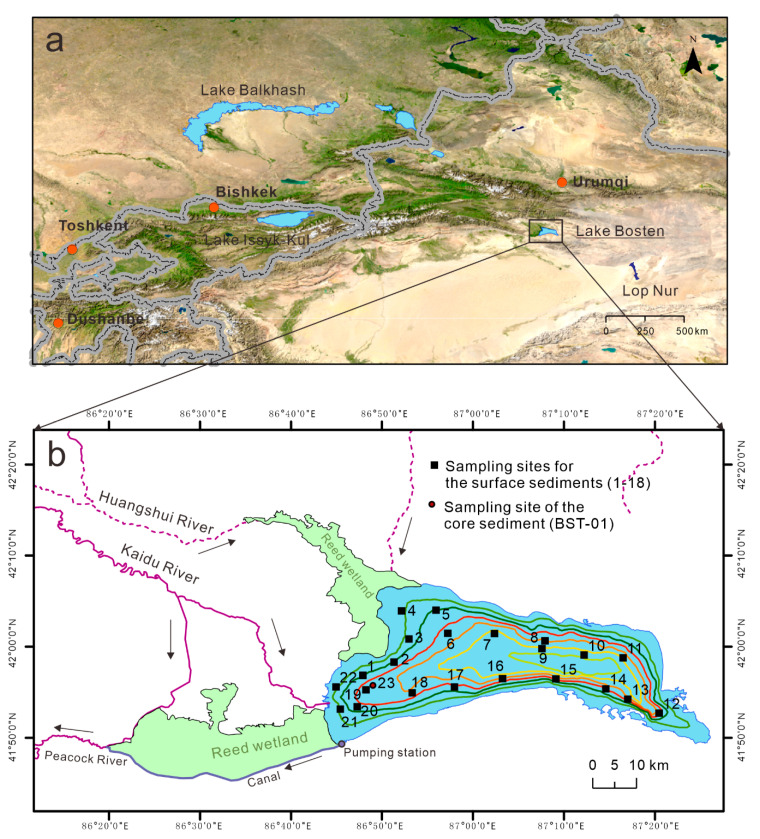
Location map of Bosten Lake (**a**) and sampling points (**b**).

Lake Bosten is the lowest point of the Yanqi Basin, located at the southern slope of the Tianshan Mountains. Its water area is vast, with an approximate length of 55 km and width of 25 km [28]. Bosten Lake is deep and dish-shaped with a flat bottom and a maximum water depth of approximately 14 m (Figure 1). The lake surface area is 1005 km^2^ and the water storage capacity is 59 × 10^8^ m^3^ [29]. The lake water is weakly alkaline and has a high hardness with a water salinity of 1.5 g/L. Lake Bosten is mainly supplied by water from the Kaidu River. This is the only river discharging water into the lake year round. The waters of Lake Bosten pass through the Peacock River in the west to Lop Nur, Tarim Basin. The annual total precipitation in the Bosten Lake basin ranges between 322 mm and 47.3 mm, and the annual evaporation ranges from 1100 to 1887 mm [30].

Because the changes in potentially-toxic elements in modern sediments (0–5 cm) were studied this reflected the possible impact of modern human activities on lake sediments. Surface sediments (0–5 cm) are the main layer of water-sediment interaction, and the layer where biological activities are relatively active [31,32,33]. The sampling protocol was mainly based on the shape of Bosten Lake and the spatial interpolation of PTEs. A disturbance-free gravity sampler (Uwitec, Mondsee, Austria) was employed to collect 22 samples from the 0–5 cm sediments of the surface layer, evenly covering the entire lake area (Figure 1 and Table S1). The 34-cm sediment core (41.92775° N, 86.81797° E) at about 7.0 m water depth (Figure 1) was sliced at 1 cm intervals in situ and analysed for the geochemical elements (Figure S1). To determine the background value of regional elements, a sediment core was retrieved from Bosten Lake (Figure 1 and Table S1). Sediment samples were placed in sequentially-marked plastic bags, while the collected surface sediment samples were freeze dried in the laboratory.

Samples (0.125 g) were collected for digestion using HCl-HNO_3_-HF-HClO_4_ microwave digestion, and certain potentially-toxic elements (Mn and V) were determined by inductively coupled plasma atomic emission spectroscopy (ICP-AES) (Prodigy, Teledyne Leeman Labs, Hudson NH, USA), while other elements were measured using inductively coupled plasma mass spectrometry (ICP-MS) (7700×, Agilent Technologies, Palo Alto, CA, USA). The error of parallel-sample analysis was <±5%. The detected limits for Mn, V, Cr, Co, Ni, Cu, Zn, As, Cd, Tl, and Pb were 0.5 mg/kg, 2 mg/kg, 0.1 mg/kg, 0.01 mg/kg, 0.05 mg/kg, 0.02 mg/kg, 0.1 mg/kg, 0.1 mg/kg, 0.01 mg/kg, 0.02 mg/kg, and 0.01 mg/kg, respectively. With the potassium dichromate volumetric method [34,35], the content of total organic carbon (TOC) was determined with analysis error of <±5%. The grain size was measured by a Mastersizer 2000 laser analyzer manufactured by Malvern, UK. Following the grain-size classification of Udden-Wentworth [36,37], the grain sizes of the sediments analyzed in this article included the clay grade (<4 μm), fine-silty grade (4–16 μm), silty grade (16–32 μm), coarse-silty grade (32–64 μm), and sandy grade (>64 μm), and the measurement error was smaller than 5%.

Bulk-rock analyses were performed using X-ray fluorescence spectrometry at ALS Chemex (Guangzhou, China). The ALS Chemex method ME-XRF26d was applied to measure Al_2_O_3_, CaO, Fe_2_O_3_, K_2_O, MgO, Na_2_O, P_2_O_5_, SiO_2_, SO_3_, and TiO_2_, and the loss-on-ignition at 1000 °C (LOI_1000_) was gravimetrically determined with the ALS Chemex method OA-GRA05x. The detection limit for the oxides and loss-on-ignition was 0.01, and the analytical precision was higher than 2% for all the oxides.

### 2.2. Pollution Evaluation Method

The potential ecological risk index (PERI) not only considered the content of heavy metals from the perspective of sedimentology, but also linked any ecological or environmental impacts with the toxicology and employed comparative and equivalence index classification techniques to assess pollution [38,39]. According to this method, the potential risk coefficient of a single element *Er* and the potential ecological risk *RI* are as follows:

(1) $$C_{f}^{i}=\left(C_{n}^{i}/B_{0}^{i}\right)$$

(2) $$E_{r}^{i}=\sum_{i=1}^{n}T_{r}^{i}\times C_{f}^{i}$$

(3) $$RI=\sum_{i=1}^{n}E_{r}^{i}$$

where *C_f_* is pollution factor of potentially-toxic element *i*, *Cn* is measured content for a single potentially-toxic element, B_0_ is the background value for potentially-toxic element, and *T_r_* is the toxicity response factor for the given potentially-toxic element, with the following values: Mn = 1 [40], V = Zn = Cr = 2, Cu = Co = Ni = Pb = 5, As = 10, Cd = 30 [41], and Tl = 10 [42]. The grading standards for the ecological risk assessment [43,44,45] are listed in Table S2.

The pollution load index (PLI) is widely applied in the pollution evaluation in soils and sediments [46,47].

(4) $$CF_{i}=\frac{C_{i}}{C_{oi}}$$

(5) $${\mathrm{PLI}}_{\mathrm{site}}=\sqrt[{n}]{{\mathrm{CF}}_{1}\times {\mathrm{CF}}_{2}\times \cdots \times {\mathrm{CF}}_{n}}$$

(6) $${\mathrm{PLI}}_{\mathrm{zone}}=\sqrt[{m}]{{\mathrm{PLI}}_{1}\times {\mathrm{PLI}}_{2}\times \cdots \times {\mathrm{PLI}}_{m}}$$

where *C_i_* is the measured content of element *I, C_oi_* is the background value of element, *CF_i_* is the pollution coefficient of element *i*, *n* is the number of evaluated elements, m is the number of sampling points in Lake Bosten, PLI_site_ is the PLI at a certain point, and PLI_zone_ is the PLI for the whole lake. The specific grading standards are divided into no pollution (PLI < 1) and moderate (1 ≤ PLI < 2), etc. [46,48].

### 2.3. Identification of the Background Values of the Elements

According to the method of Palaeoecological Investigation of Recent Lake Acidification (PIRLA) proposed by Binford (1990) [49] to calculate the elemental background value of a sedimentary section, the specific operation is conducted as follows [50]: first, the relatively-stable average value (X) and standard deviation (SD) of the element content at the bottom of the sediment core are calculated. If the element content of the next upper layer of the sediment core is lower than X + SD, then this part of the element content is included in the relatively-stable average value, and the average value (X) and SD are recalculated. The operation is repeated until the element content of the next part is higher than X + SD. The relatively-stable average value obtained by this method could be regarded as the background value of the element.

### 2.4. Statistical Methods

Moran’s I was adopted to reveal the spatial relevance relationship among the PTEs in the neighborhood set at each location [51]. Moran’s I = 0, suggests a random spatial distribution, at −1 ≤ Moran’s I < 0, there is a negative correlation, while 0 < Moran’s I ≤ 1 suggests a positive correlation [52,53]. Moran’s I testing was conducted using GeoDa software version 1.14.0 24 [54]. With the software NCSS 12.0 (test version), the two-way dendrogram generated with the method of two-way cluster analysis [55] was employed to reveal the statistical similarity among the clustering results of the potentially-toxic elements and sample sites. Redundancy analysis (RDA) was used to reveal the possible sources of the PTEs and their influencing factors. RDA examines the changes in PTEs along a specific gradient (whole rock composition and grain size) and is currently the most widely applied environmental factor analysis tool in the field of environment and ecology [56,57,58,59]. RDA was conducted by Canoco 5 [60] with none data transformation and unrestricted permutations (number of permutations is 499).

## 3. Results

**Figure 2:**
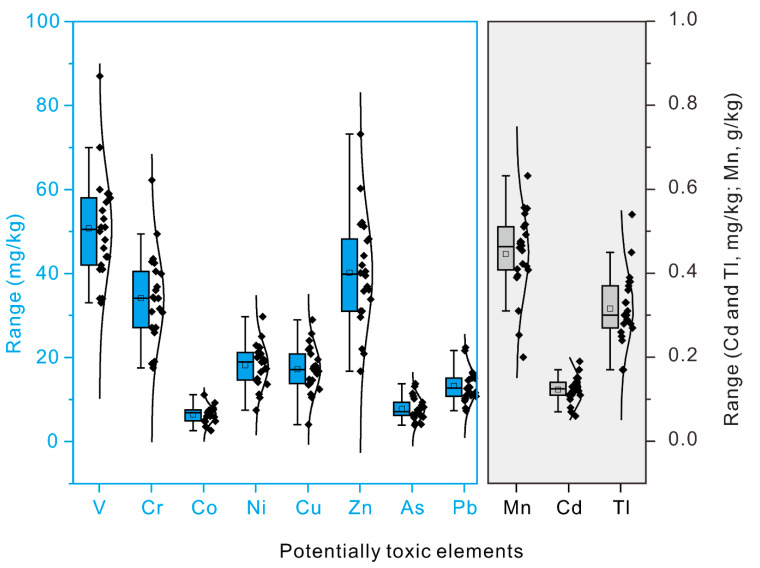
Statistical characteristics of the potentially-toxic elements (PTEs) in the Bosten Lake surface sediments. The plots for PTEs in black box and transparent box were used different vertical coordinates.

Statistical characteristics of the latent PTEs, particle sizes, whole-rock composition, and organic carbon content are shown in Table S3. The statistical characteristics of the PTEs in the surface sediments are shown in Figure 2. The highest content of the PTEs was that of Mn. The Mn content was between 0.20 and 0.63 g/kg. The lowest average contents of the PTEs were those of Cd and Tl. The Cd content was between 0.06 and 0.19 mg/kg and the Tl was between ~0.17 and 0.54 mg/kg (Figure 2). The variations of PTEs in the sediment core are shown in Figure S1. The background values were obtained according to the above method of identifying the background values of the elements. The background values for PTEs were extracted with the PIRLA method, and the background values for Mn, V, Zn, Cr, Co, Ni, Cu, As, Cd, Tl, and Pb were 0.33 g/kg (Mn), 37.49 mg/kg (V), 35.52 mg/kg (Zn), 23.18 mg/kg (Cr), 4.81 mg/kg (Co), 13.32 mg/kg (Ni), 12.14 mg/kg (Cu), 5.70 mg/kg (As), 0.10 mg/kg (Cd), 0.22 mg/kg (Tl), and 8.19 mg/kg (Pb).

**Figure 3:**
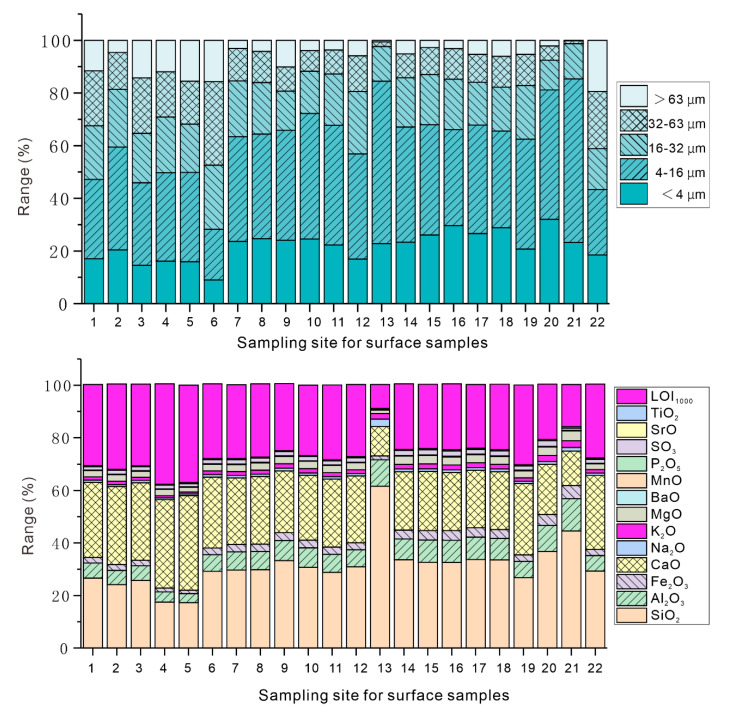
The grain size composition (upper) and bulk-rock analysis (lower) results for the surface sediments of Lake Bosten.

The grain size compositions for the surface sediments are shown in Figure 3. Following the grain-size classification of Udden-Wentworth [36,37], the surface sediments of Lake Bosten were classified into five grain size grades (Figure 3): clay grade (<4 μm), fine-silty grade (4–16 μm), silty grade (16–32 μm), coarse-silty grade (32–64 μm), and sandy grade (>64 μm). The highest average content was that of fine silt (4–16 μm). The fine silt content ranged from 19.21–62.07%. The lowest content was that of sand (>64 μm), with a range of 0.16–19.40%. The TOC ranged from 4.46 to 48.36 g/kg, with an average value of 32.57 g/kg (Table S3).

**Figure 4:**
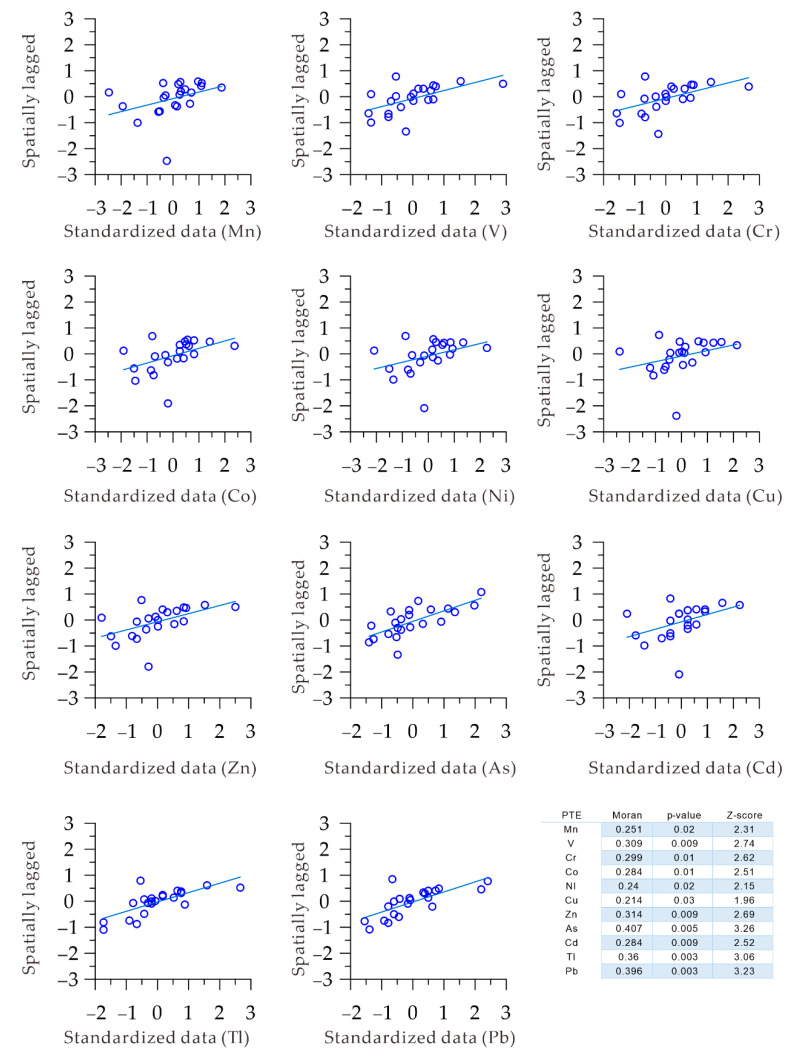
Moran’s I plots for the PTEs and statistic test results for Moran’s I.

The mathematical statistics could not reflect the differences in the spatial distribution among the various potentially-toxic elements, and it was impossible to determine the geographical connotations, leading to a lack of relevance for the ecological protection of the lake. The scatter plots of Moran’s I suggested that there are significant spatial positive correlations for the PTEs in the surface sediments (Figure 4). A positive Moran’s I implies that as the spatial distribution position (distance) decreases, the correlation becomes more significant. In the region of Lake Bosten, the positive value of Moran’s I suggested that the correlation becomes more significant with increasing aggregation of the spatial distribution position. Through spatial autocorrelation analysis (Figure 4), it was found that spatial autocorrelation occurs among the potentially-toxic elements, and the spatial distribution map of these potentially-toxic elements could be established by the inverse distance spatial interpolation method [61].

## 4. Discussion

### 4.1. The Possible Influencing Factors for the PTEs in Bosten Lake Sediments

The content of PTEs in the surface sediments of Bosten Lake reflects the distinctive characteristics of lake sediments in arid regions, similar to Lake Balkhash in Kazakhstan [62], and Lakes Chaiwopu [63], Ebinur [64], and Sayram [65] in Xinjiang, China. However, compared to humid lakes, such as Lakes Taihu [66], Chaohu [67], and Erhai [68], the LOI_1000_ levels of the Bosten Lake sediments are higher and the contents of Al_2_O_3_, Fe_2_O_3_, and other potentially-toxic elements are lower. Bosten Lake is located in the arid area of central Asia. On the one hand, the bedrock of the lake watershed contains many carbonates and evaporative salts, and the saturation index of aragonite and calcite of its surface water body indicates supersaturation [16]. On the other hand, regional evaporation is beneficial to the enrichment of authigenic carbonate minerals, leading to the dilution of potentially-toxic elements in the lake sediments.

**Figure 5:**
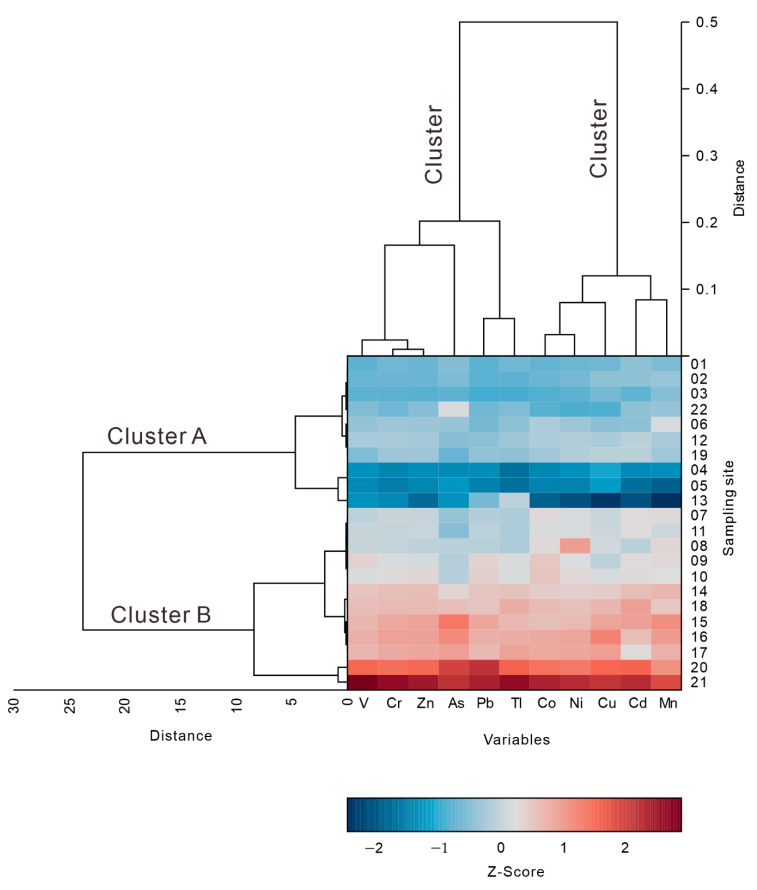
Two-way clustering results for the potentially-toxic elements and sampling sites of the surface sediments.

**Figure 6:**
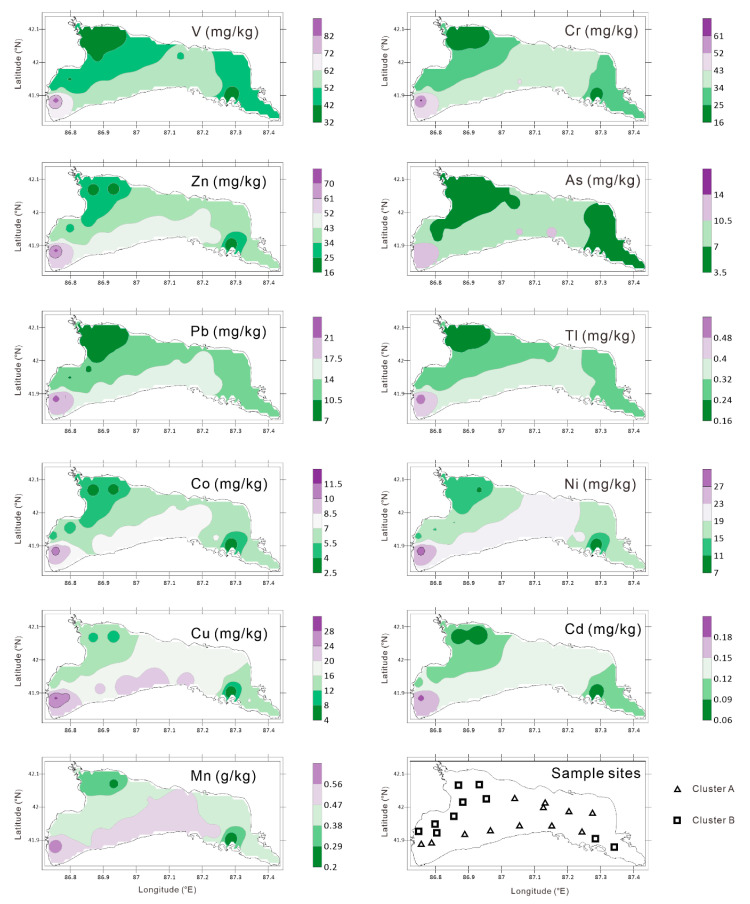
The spatial distribution of the PTEs and two-way cluster results of the sampling sites (lower left part) for the surface sediments of Lake Bosten.

The two-way clustering dendrogram revealed the relative similarity as reflected by the standardized Euclidean distance between the sampling sites and potentially-toxic elements. As indicated by the similarities among the sampling sites, the sampling sites could be clearly divided into two categories with a threshold of 10—clusters A and B (Figure 5 and Figure 6, respectively). The sampling points located in cluster A were mainly distributed in the middle of the lake and presented a continuous distribution (Figure 6) and were characterized by a low content of the potentially-toxic elements (Figure 5). The sampling points located in cluster B were mostly distributed in the east and west of the lake (Figure 6) and were characterized by a high content of PTEs (Figure 5). Similar to the sampling horizons, the two-way clustering dendrogram is an effective tool to reveal the similarity relationship among the potentially-toxic elements. The potentially-toxic elements can be divided into two groups with threshold of 0.3—clusters I and II. Cluster I includes V, Cr, Zn, As, Pb, and Tl, and cluster II includes Co, Ni, Cu, Cd, and Mn. These two types of potentially-toxic elements may have differences in their material sources.

On the one hand, the accuracy of the spatial distribution map is related to the spatial interpolation method, but the more important reason should be the sampling density. The spatial distribution map of potentially-toxic elements can only show a general trend of potentially-toxic elements in the sediments of Lake Bosten. The spatial distribution map (Figure 6) clearly shows that the low-value areas of the potentially-toxic elements are primarily distributed on the northwest and southeast sides of the lake area. High-value areas occur in the middle and southwest of the lake area, with the highest content observed on the southwest side. This may be related to the hydrodynamic conditions of the lake. The southwest side hosts the main entrance and exit of the lake, while the east side occurs at the end of the lake water cycle with a calm water deposition environment.

**Figure 7:**
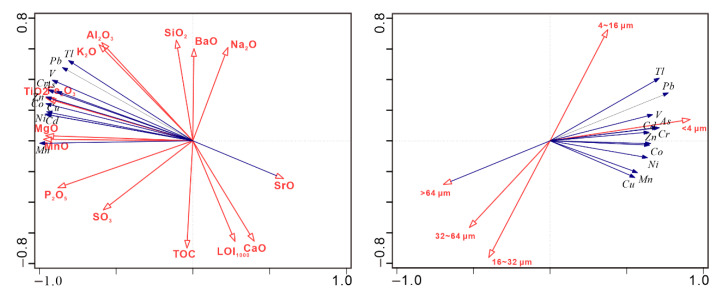
Redundancy analysis of the correlation among the potentially-toxic elements, bulk-rock composition, total organic carbon (TOC), and components of the different grain sizes. For the left-hand diagram, the two axes account for 99.95% of total eigenvalues (axis 1: 99.38% and axis 2: 0.57%) with permutation test results (pseudo-F = 134, P = 0.002). For the right-hand diagram, the two axes account for 99.98% of total eigenvalues (axis 1: 98.86% and axis 2: 1.12%) with permutation test results (pseudo-F = 2.9, *p* = 0.052).

The material composition, structure, and organic matter content in the sediments affect the content and distribution of the potentially-toxic elements. The bulk-rock composition reflects the material composition characteristics of the sediments, while the particle size reflects their structural characteristics. To determine the factors that influence the element content among the environmental variables, the RDA method was considered to compare the correlations among the potentially-toxic elements, bulk-rock composition, total organic carbon (TOC), and components of different grain sizes (Figure 7). Existing research has indicated that a high organic matter content may increase the adsorption of pollutants in the water environment [69]; however, in Lake Bosten, the relationship with the organic carbon is obviously different. The content of the PTEs is related to Al_2_O_3_, K_2_O, Fe_2_O_3_, TiO_2_, MgO, and MnO. This implies that the PTEs in the Bosten Lake sediments mainly comprise silicate and Fe–Mn minerals. Moreover, this also indicates that the content of the PTEs is weakly influenced by the organic matter. In addition, the PTEs, clay grade (<4 μm), and fine-silty grade (4–16 μm) showed significant correlations, and the silty grade (16–32 μm), coarse-silty grade (32–64 μm), and sandy grade (>64 μm) were positively related to the first axis, which revealed that the storage medium of the PTEs mainly consists of small particles with a grain size <16 μm.

### 4.2. Potential Ecological Risks of the PTEs

**Figure 8:**
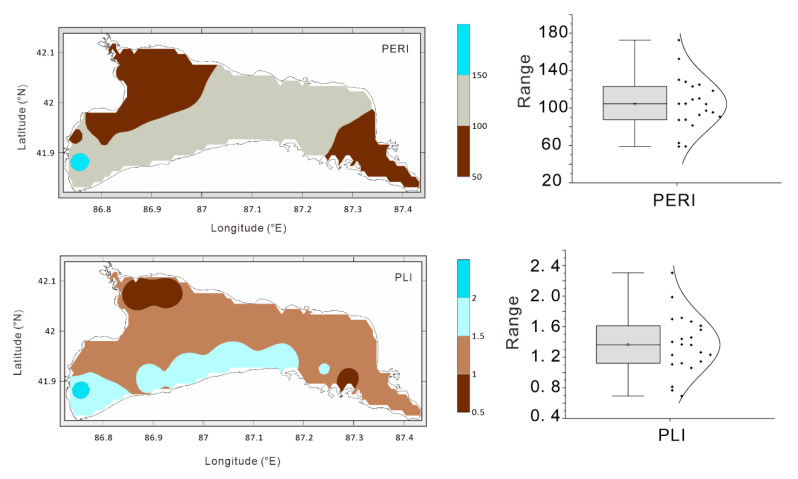
The pollution assessment of PTEs in the surface sediments of Lake Bosten.

The spatial distribution map of the PERI and PLI due to the PTEs in the lake sediments of Lake Bosten revealed, on the one hand, spatial differences of the pollution for PTEs, and on the other hand, it reflects future targeted lake ecological protection by the government and the public. Based on the grading standards of the ecological risk assessment index (Table S2), a PERI lower than 150 indicates a low ecological risk, while a PERI above 150 indicates a moderate ecological hazard. Figure 8 shows that the ecological risk caused by the potentially-toxic elements in Bosten Lake is low, and only one of the 22 sampling sites (site 21) exhibited a PERI higher than 150. Based on the PLI, among the 22 sampling points, three points have a PLI_site_ value lower than 1, indicating no pollution. The PLI_site_ value of 18 points is between 1 and 2, reflecting moderate pollution, and only one sample point has a value higher than 2, indicating heavy pollution. Combined with the spatial distribution map of PLI_site_, the area with a PLI_site_ value higher than 1 accounts for 87.2% of the total lake area. By calculating the overall pollution index of Lake Bosten, it is found that the PLI_zone_ value of the Lake Bosten sediments due to the potentially-toxic elements is 1.31, which indicates that the Lake Bosten sediments are moderately polluted as a whole. In general, the pollution of PTEs in the Bosten Lake sediments is low, but due to the trace toxicity and long-term accumulation properties of these potentially-toxic elements, the lake pollution problem still requires attention. It must be mentioned that two different methods for evaluation resulted in some differences of the pollution risk, which were mainly related to the evaluation criteria and thresholds. Although the methods we used were widely used in the study of the health risk assessment of potential toxic elements in sediments from China [42,70] and other countries [71,72]. The evaluation method does have the problems of the practicality of evaluation criteria in the study area. At present, studies on the toxicological effects of Bosten Lake sediments have not been carried out, so it is not possible to propose evaluation methods and standards applicable to this research area for Bosten Lake. 

As the water outflow of Lake Bosten is controlled by a man-made pumping station (Figure 1), the circulation of the lake is not smooth. Most of the pollutants in the watershed will be deposited into the sink of Lake Bosten. Through the research in this paper, it was indeed found that there is an enrichment of potential toxic elements in the surface sediments. Therefore, to control the enrichment of PTEs in the lake, the most fundamental thing is to reduce the emission of pollution sources including industrial, agricultural, and domestic sewage discharge. This should be a high priority for the government and the public in order to avoid serious pollution problems in the future.

## 5. Conclusions

In this paper, the influence of human activities on the enrichment of PTEs in lake sediments of underdeveloped areas in China was revealed, and an approach to carrying out research on the influence factors of PTEs with the data of the grain size, bulk-rock composition, and organic matter content in lake sediments was provided. The following conclusions were obtained:

(1) The enrichment of authigenic carbonate minerals leads to the dilution of the PTEs in the lake sediments of arid regions. All PTEs (Mn, V, Cr, Co, Ni, Cu, Zn, As, Cd, Tl, and Pb) exhibit significant spatial autocorrelation. The low-value areas for PTEs are primarily distributed on the northwest and southeast sides of the lake area. The high-value areas occur in the middle and southwest of the lake area.

(2) Use of data including whole rock, and grain size of lake sediments is an effective way to study the influencing factors of potential toxic elements. The content of the PTEs is related to Al_2_O_3_, K_2_O, Fe_2_O_3_, TiO_2_, MgO, and MnO, suggesting that the PTEs primarily comprise silicate and Fe–Mn minerals in the Bosten Lake sediments, and the storage medium of these potentially-toxic elements mainly consists of small particles with a grain size <16 μm. 

(3) The enrichment of PTEs in modern lake sediments under the background of enhanced human activities was revealed in this paper. The PERI suggests that the ecological risk in Bosten Lake is low, and the PLI shows the surface area with PLI higher than 1 and a moderate pollution level accounts for 87.2% of the total lake area. The overall PLI of Lake Bosten due to the potentially-toxic elements is 1.31, suggesting a moderate pollution level.

## Supplementary Materials

The following are available online at https://www.mdpi.com/2305-6304/8/3/77/s1: Figure S1, The values for the potentially-toxic elements in the core sediment of Lake Bosten; Table S1, Geographical coordinates of sampling points; Table S2, Grading standards for ecological risk assessment index; Table S3, Statistical characteristics of latent heavy metal elements, particle size, whole rock composition, and organic carbon content.
