# Supplementary material for: Spatial Distribution and Ecological Risks of the Potentially-Toxic Elements in the Surface Sediments of Lake Bosten, China
Source: Toxics. 2020 Sep 22;8(3):77. doi: 10.3390/toxics8030077 (PMC7560408; doi:10.3390/toxics8030077)
Supplement: Supplementary file 1 [file toxics-08-00077-s001.pdf]

Supplementary Materials: Spatial Distribution and Ecological Risks of the Potentially-Toxic Elements in the Surface Sediments of Lake Bosten, China

Long Ma, Jilili Abuduwaili and Wen Liu

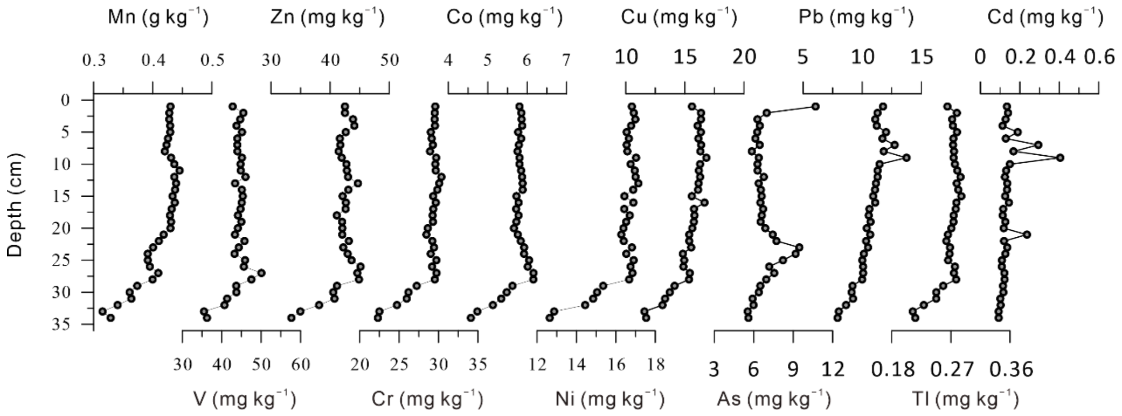

Figure S1. The values for the potentially toxic elements in the core sediment of Lake Bosten.

Table S1. Geographical coordinates of sampling points.

Sampling site.	Sediment type	Longitude (°E)	Latitude (°N)
1	surface	86.79850	41.94835
2	surface	86.85553	41.97216
3	surface	86.88277	42.01477
4	surface	86.86965	42.06614
5	surface	86.93248	42.06765
6	surface	86.95416	42.02490
7	surface	87.03997	42.02465
8	surface	87.13220	42.01129
9	surface	87.12680	41.99708
10	surface	87.20416	41.98515
11	surface	87.27553	41.98031
12	surface	87.34146	41.87899
13	surface	87.28428	41.90440
14	surface	87.24369	41.92354
15	surface	87.15228	41.94183
16	surface	87.05464	41.94198
17	surface	86.96651	41.92712
18	surface	86.88876	41.91617
19	surface	86.80401	41.92196
20	surface	86.78840	41.89051
21	surface	86.75706	41.88649
22	surface	86.74919	41.92677
23	core	86.81797	41.92775

Table 2. Grading standards for ecological risk assessment index

E_r^i	Description	$PERI$	Description
$E_r^i < 40$	Low ecological risk	$PERI < 150$	Low risk
$40 \leq E_r^i < 80$	Moderate ecological risk	$150 \leq PERI < 300$	Moderate ecological risk
$80 \leq E_r^i < 160$	Considerable ecological risk	$300 \leq PERI < 600$	Considerable ecological risk
$160 \leq E_r^i < 320$	High ecological risk	$PERI \geq 600$	Very high ecological risk
$E_r^i > 320$	Very high ecological risk	–	–

Table S3. Statistical characteristics of latent heavy metal elements, particle size, whole rock composition, organic carbon content.

Composition	Mean	Standard Deviation	SE of mean	Coefficient of Variation	Minimum	Median	Maximum
Mn (g/kg)	0.45	0.10	0.02	0.22	0.20	0.46	0.63
V (mg/kg)	50.77	12.51	2.67	0.25	33.00	50.50	87.00
Cr (mg/kg)	34.15	10.55	2.25	0.31	17.50	34.05	62.20
Co (mg/kg)	6.39	1.99	0.42	0.31	2.60	6.80	11.10
Ni (mg/kg)	18.11	5.13	1.09	0.28	7.40	18.95	29.70
Cu (mg/kg)	17.19	5.53	1.18	0.32	4.00	17.05	29.00
Zn (mg/kg)	40.20	13.19	2.81	0.33	16.70	39.80	73.20
As (mg/kg)	7.73	2.72	0.58	0.35	3.90	7.05	13.70
Cd (mg/kg)	0.12	0.03	0.01	0.24	0.06	0.13	0.19
Tl (mg/kg)	0.32	0.08	0.02	0.27	0.17	0.30	0.54
Pb (mg/kg)	13.21	3.82	0.81	0.29	7.30	12.70	22.40
<4μm (%)	21.86	5.47	1.17	0.25	9.03	23.00	32.04
4~16μm (%)	40.05	9.98	2.13	0.25	19.21	39.84	62.07
16~32μm (%)	18.34	3.38	0.72	0.18	11.29	18.95	24.35
32~63μm (%)	12.74	6.90	1.47	0.54	1.11	11.78	31.80
>63μm (%)	7.01	5.41	1.15	0.77	0.16	5.22	19.40
Median size (μm)	12.51	5.71	1.22	0.46	6.45	10.07	30.25
TOC (g/kg)	32.57	8.82	1.88	0.27	4.46	33.71	48.36
SiO_2 (%)	31.32	8.94	1.91	0.29	17.27	30.27	61.57
Al_2O_3 (%)	7.21	2.02	0.43	0.28	3.46	6.98	12.32
Fe_2O_3 (%)	2.81	0.89	0.19	0.32	1.32	2.80	4.97
CaO (%)	24.75	5.70	1.21	0.23	11.20	25.35	36.00
Na_2O (%)	1.04	0.43	0.09	0.41	0.61	0.97	2.82
K_2O (%)	1.57	0.43	0.09	0.27	0.76	1.53	2.62
MgO (%)	2.65	0.50	0.11	0.19	1.26	2.69	3.68
BaO (%)	0.05	0.01	0.00	0.12	0.04	0.05	0.06
MnO (%)	0.06	0.02	0.00	0.24	0.03	0.06	0.09
P_2O_5 (%)	0.11	0.02	0.00	0.19	0.05	0.12	0.13
SO_3 (%)	1.54	0.41	0.09	0.26	0.33	1.54	2.21
TiO_2 (%)	0.32	0.08	0.02	0.26	0.17	0.31	0.54
LOI_{1000} (%)	26.89	6.08	1.30	0.23	9.74	27.49	38.19

© 2020 by the authors. Licensee MDPI, Basel, Switzerland. This article is an open access article distributed under the terms and conditions of the Creative Commons Attribution (CC BY) license (http://creativecommons.org/licenses/by/4.0/).
